# Micronutrients decline under long-term tillage and nitrogen fertilization

**DOI:** 10.1038/s41598-019-48408-6

**Published:** 2019-08-19

**Authors:** Santosh Shiwakoti, Valtcho D. Zheljazkov, Hero T. Gollany, Markus Kleber, Baoshan Xing

**Affiliations:** 1 Department of Crop and Soil Sciences, Washington State University, Pullman, WA 99164 USA; 2 Department of Crop and Soil Science, 3050 SW Campus Way, Oregon State University, Corvallis, OR 97331 USA; 3 USDA Agricultural Research Service, Columbia Plateau Conservation Research Center, 48037 Tubbs Ranch Rd., Adams, OR 97810 USA; 4 Stockbridge School of Agriculture, University of Massachusetts, Amherst, MA 01003 USA

**Keywords:** Plant ecology, Chemistry

## Abstract

Tillage and nitrogen (N) fertilization can be expected to alter micronutrient dynamics in the soil and in plants over time. However, quantitative information regarding the effects of tillage and N application rates on micronutrient dynamics is limited. The objectives of this study were (a) to determine the long-term effect of different tillage methods as well as variation in N application rates on the distribution of Mehlich III extractable manganese, copper, zinc, boron, and iron in soils and (b) to assess accumulation of the same nutrients in wheat (*Triticum aestivum* L.) tissues. The system studied was under a dryland winter wheat-fallow (WW-F) rotation. Tillage methods included moldboard (MP), disk (DP) and sweep (SW), and the N application rates were 0, 45, 90, 135, and 180 kg ha^−1^. The concentration of soil manganese was greater under DP (131 mg kg^−1^) than under MP (111 mg kg^−1^). Inorganic N application reduced extractable soil copper while, it increased manganese accumulation in wheat grain over time. Comparison of micronutrients with adjacent long-term (since 1931) undisturbed grass pasture revealed that the WW-F plots had lost at least 43% and 53% of extractable zinc and copper, respectively, after 75 years of N fertilization and tillage. The results indicate that DP and inorganic N application could reduce the rate of micronutrient decline in soil and winter wheat grain over time compared to MP and no N fertilization.

## Introduction

Nitrogen fertilization plays a significant role in the dynamics of soil organic matter (SOM). Most of the micronutrients are largely SOM bound and will be released when SOM decomposition is stimulated^1,2^. The decomposition of SOM is stimulated by tillage through changes in soil water, aeration, temperature, and nutritional environment^3,4^. No-tillage or reduced tillage accumulates SOM in the upper surface whereas SOM are uniformly mixed to a plow depth under a conventional tillage. The stratification of SOM can lead to varying distribution of micronutrients in soil profile and mislead the farmers on determining the optimum fertilizer application rate^5^. Therefore, understanding the role of tillage and N fertilization in the availability and distribution of micronutrients is crucial in formulating and developing cropping system strategies for sustainable agriculture.

Micronutrient availability in cultivated plots is affected by tillage methods^6^. It has been reported that even a slight soil disturbance or tillage increases chemical and microbial activity that enhances nutrient release via mineralization of OM^7^. There have been inconsistencies in research reporting tillage effects on the concentration of extractable iron (Fe), zinc (Zn), copper (Cu), and manganese (Mn). Mahler^6^ observed higher extractable Fe and Mn under conventional and reduced tillage than under no-tillage in the soils of northern Idaho, whereas other researchers reported the opposite^1,8,9^. Lavado *et al*.^5^ and Hickman^10^ reported concentrations of extractable soil Cu and Zn were unaffected by tillage. However, Shuman and Hargrove^11^ observed lower Mn and Fe under no-tillage and reduced tillage than under conventional tillage due to a shift in exchangeable forms of Mn and Fe from inorganic to organic forms. Under reduced or no-tillage, the availability of some micronutrients increases and they appear in more readily available forms in the surface soils due to metal complexation by OM^12^. Additionally, OM increases microbial exudates which have been reported to enhance micronutrient availability to plants, especially Fe^13^, Cu, Mn, and Zn^14^. A similar effect of OM on B has been reported by Sarkar *et al*.^15^.

Another key factor for micronutrient availability in plants and soils is N fertilization. The application of N fertilizers is largely based on crop demands while the input of micronutrients, which are significantly impacted by N fertilization rates, is less common. It has been reported that increasing N supply can enhance accumulation of Zn and Fe in wheat grain^16^. High N supply increases transporter proteins and nitrogenous chelators involved in the uptake, translocation, remobilization and grain allocation of Fe and Zn, and hence, increases the Zn and Fe in wheat grain^17^. However, Cakmak *et al*.^18^ reported a decline in Zn and Fe in wheat grain with high N fertilization rates. Inconsistency in results from varying N application rates on soil micronutrient dynamics were also reported previously. A study by Wang *et al*.^17^ indicated that N fertilization significantly increased the availability of Cu, Mn, and Fe and attributed it to the nitrification derived acidity. Contrastingly, Malhi *et al*.^19^ reported that high N fertilization rate decreased the concentrations of extractable Cu and Zn and suggested further investigation is required to determine the cause.

Information on the dynamics of plant essential micronutrients as a function of tillage and N application rates is limited, inconsistent, and region specific. Therefore, there is a need to examine the impact of N application rates and tillage methods on the concentration of micronutrients in soil and plant. This study was undertaken with the objective to investigate the long-term (75 years) effects of tillage and N fertilization rates on Mn, Cu, B, Fe, and Zn in soil, and wheat grain and straw under dryland WW-F rotation in the Pacific Northwest (PNW). The conceptual approach consisted of analyzing the soil (four depths: 0–10, 10–20, 20–30, and 30–60 cm) and wheat (grain and straw) samples of 1995, 2005 and 2015 for Mn, Cu, B, Fe, and Zn, and comparing the analyzed micronutrients among the treatments to determine the 20-years trend of micronutrient dynamics. Additionally, we compared the soil micronutrients of WW-F plots with that of nearby long-term (since 1931) undisturbed grass pasture (GP) plots to detect the tillage and N fertilization induced changes in soil micronutrients during 75 years. We hypothesized that: (i) The concentrations of extractable micronutrients are greater under conservation tillage [disk plow (DP) and sweep (SW)] than under conventional tillage [moldboard plow (MP)] over time. The basis of this hypothesis is that the greater amount of crop residue left at the surface due to reduced soil disturbance under SW and DP will allow accumulation of more SOM than under MP, noting soil OM is a source of plant essential micronutrients, and; (ii) The plots with high N application rates will have a greater concentration of micronutrients than in the zero or low N application rate plots over time. This hypothesis is based on the well-established fact that the N increases organic matter through increased crop root biomass and releases micronutrients in soil upon the decomposition of OM. Furthermore, N fertilization increases soil acidity and the availability of micronutrients in soil increase under acidic conditions.

## Results

Only the significantly (*p* ≤ *0*.*05*) affected micronutrients are reported in the text below.

### Tillage effect on soil micronutrients

**Figure 1:**
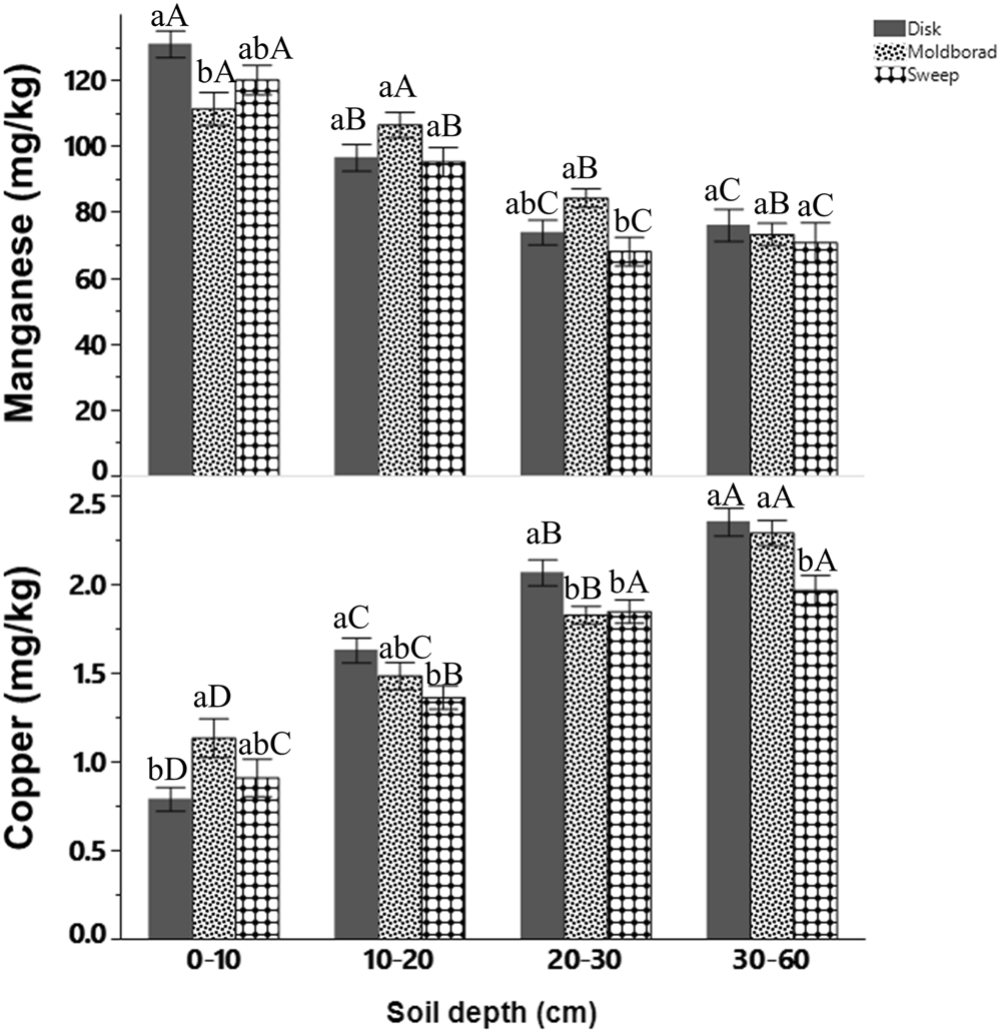
Mehlich III extractable manganese (top) and copper (bottom) as influenced by the interaction of tillage system and soil depth in 2015. Bars sharing the same letters are not significantly different at 0.05 probability level. Lowercase letters are comparison of tillage system within each soil depth, and uppercase letters are comparison of tillage system across the four soil depths.

The concentration of Mehlich III extractable Mn was significantly (*P* < 0.01) affected by the tillage method × soil depth interaction (Table 1). The DP had greater extractable Mn (131 mg kg^−1^) than under MP (111 mg kg^−1^) while no significant differences in extractable Mn were found between SW (121 mg kg^−1^), DP or MP at the 0–10 cm soil depth (Fig. 1). In the 20–30 cm depth, the MP had similar extractable Mn (84 mg kg^−1^) to that under DP (73 mg kg^−1^) but had greater extractable Mn than under SW (68 mg kg^−1^) (Fig. 1). Extractable Mn under DP and SW significantly declined with soil depth, while no significant decline in extractable Mn was observed under MP beyond 10–20 cm (Fig. 1).

**Table 1:** ANOVA table for the main and interaction of tillage, depth, and N rate effects on the concentration on Mehlich III extractable manganese (Mn), copper (Cu), zinc (Zn), iron (Fe), and Boron (B).

Source of variation	Manganese	Copper	Zinc	Iron	Boron
Tillage (T)	0.12	<0.01	0.05	0.61	0.44
Depth (D)	<0.01	<0.01	0.17	**<0.01**	0.96
N rate (N)	0.94	**<0.01**	0.06	0.78	0.68
T x D	**0.01**	**<0.01**	0.88	0.44	1.11
N x D	0.68	0.91	0.87	0.67	1.01
T x N	0.81	0.09	0.06	0.97	0.58
T x N x D	1.0	0.87	0.07	1.01	1.13

Significant effects (p-value < 0.05) that require multiple means comparison are in bold.

**Figure 2:**
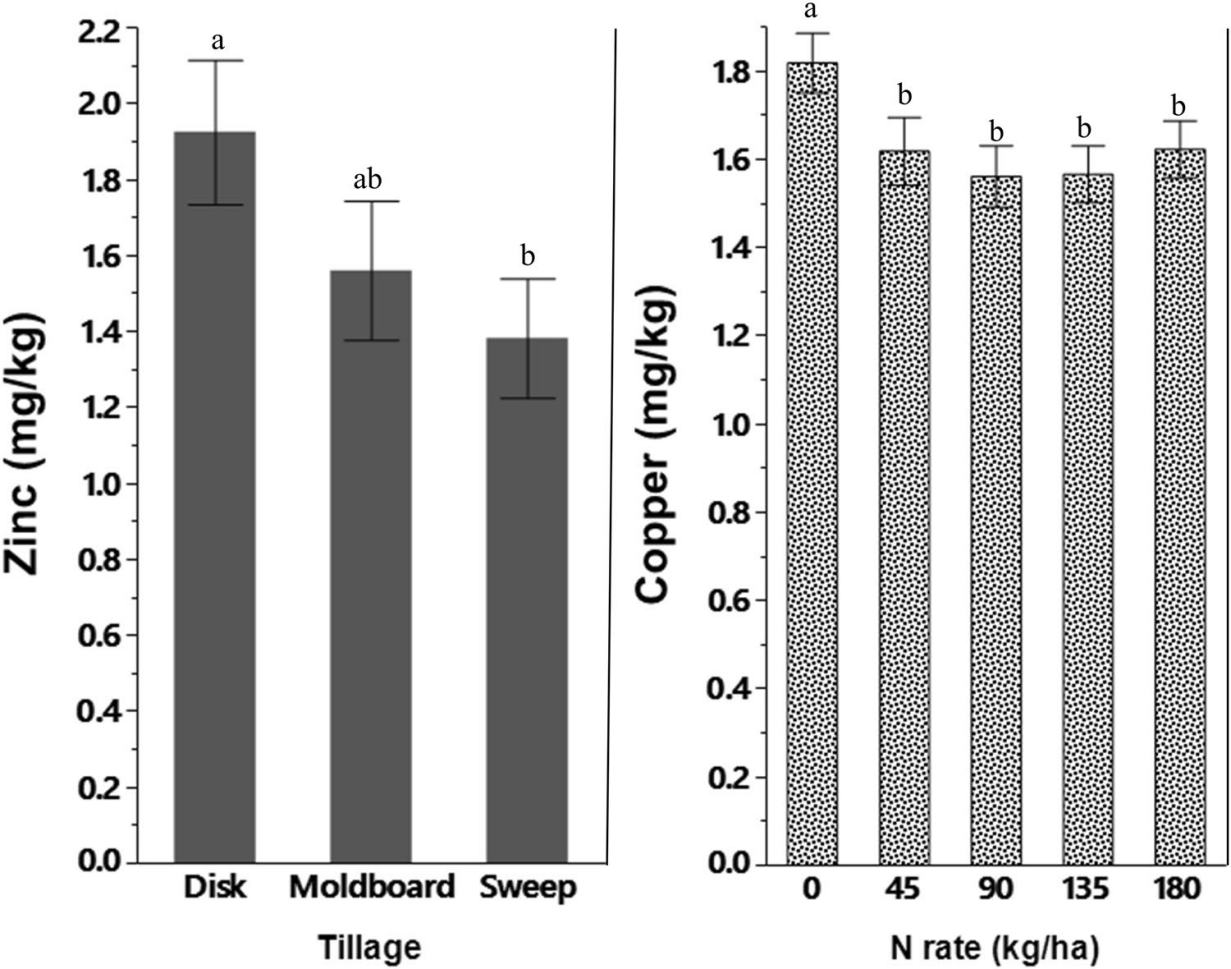
Mehlich III extractable soils zinc (left) and copper (right) in the top 10 cm soil depth as influenced by tillage system and N application rates in 2015, respectively. Bars sharing the same letters are not significantly different at 0.05 probability level.

The MP had greater extractable Cu than under DP (1.13 mg kg^−1^ vs. 0.79 mg kg^−1^) in the 0–10 cm soil depth, and extractable Cu increased with soil depth under all tillage systems (Fig. 1). Concentrations of extractable Cu under DP were 0.79, 1.63, 2.06, and 2.35 mg kg^−1^ at the 0–10, 10–20, 20–30, and 30–60 cm soil depths, respectively (Fig. 1). The concentration of extractable Zn was also affected by the tillage methods; Zn was significantly greater under DP (1.92 mg kg^−1^) than under SW (1.38 mg kg^−1^) while it was comparable to Zn under MP (1.56 mg kg^−1^) (Fig. 2).

### Nitrogen fertilization effect on soil micronutrients

In this study, only extractable Cu was found to be affected by N fertilization rates. Extractable Cu was significantly greater without N application and declined with the application of N fertilizer (Fig. 2). A three-way interaction of N rate, tillage and year were observed for extractable Zn, Fe, and B in this study (Table 2). However, we did not observe any consistent trend of micronutrients change over the time.

**Table 2:** Three-way interaction effect of tillage system, N application rate, and year on extractable zinc, iron, and boron in the soil.

N rate	1995			2005			2015		
	MP^†^	SW	DP	MP	SW	DP	MP	SW	DP
(kg ha^−1^)	Zinc (mg kg^−1^)								
0	0.14a^‡^	0.48a	0.40a	4.95a	4.61a	5.36a	0.48a	0.90a	0.54a
45	0.14a	0.42a	0.34a	5.52a	2.31b	3.45ab	0.47a	0.41a	0.46a
90	0.32a	0.37a	0.31a	4.50b	3.44b	7.32a	0.34a	0.35a	0.77a
135	0.16a	0.23a	0.51a	3.39ab	4.89a	2.01b	0.72a	0.28a	0.72a
180	0.09a	0.34a	0.38a	1.90b	1.31b	5.96a	0.20a	0.32a	0.64a
	Iron (mg kg^−1^)								
0	142a	151a	147a	316a	337a	364a	145a	138a	128a
45	143a	140a	157a	350a	382a	268b	141a	156a	166a
90	163a	143a	126a	341a	352a	341a	162a	152a	139a
135	153a	161a	165a	342a	310a	302a	167a	181a	173a
180	164a	172a	157a	330a	293a	320a	192a	199a	181a
	Boron (mg kg^−1^)								
0	8.52a	9.01a	9.02a	8.56a	8.48a	8.49a	2.19b	2.36ab	2.51a
45	8.78a	8.12a	7.73b	13.11a	8.85b	8.41b	2.80a	2.13c	2.51b
90	8.37a	8.33a	8.56a	8.96a	8.79a	7.98b	2.47b	2.27b	2.77a
135	8.84a	8.63a	8.99a	8.44a	8.47a	8.49a	2.26b	2.21b	2.61a
180	8.69a	8.10b	8.28b	8.06a	8.26a	8.28a	2.17b	2.08b	2.36a

^†^MP: moldboard plow; DP: disk plow; and SW: sweep.

^‡^Means followed by the same letter in a row indicate no significant differences between tillage system within each year at 0.05 probability level.

### Soil micronutrients after 75 years of N fertilization and tillage versus grass pasture (GP)

Since DP was the best tillage in maintaining micronutrients compared to the other tillage treatments in this study, we compared the soil micronutrients of DP plots with that of GP plots, the reference/baseline of this study, to detect the treatment’s effect over 75 years.

Compared with the nutrients in the GP plots, extractable Zn and Cu concentrations in the cultivated plots declined more than other nutrients. Extractable Zn decreased by at least 43%, and Cu decreased by 53% in the top 10 cm soil depth compared with their respective concentrations in the GP plots (Table 3). The concentrations of Mn and Zn at the 20–30 cm and 30–60 cm soil depths were comparable to those of GP. At the 20–30 cm soil depth, N fertilization contributed to a decrease in extractable Cu when compared with GP. Similarly, a pronounced decrease in extractable Cu was found at the 30–60 cm soil depth of DP plots that received N fertilizer above 45 kg ha^−1^. Iron was not tested in the soils of GP, and B was detected only at the 20–30 cm depth in GP.

**Table 3:** Impact of 75 years of inorganic N application rate (N rate) on soil micronutrients and soil pH of dryland winter wheat-fallow cropping system under disk tillage management compared to nearby undisturbed grass pasture (GP).

Nutrients	Soil depth (cm)	N rate (kg ha^−1^)					GP	Cultivation effect^‡^
		0	45	90	135	180		
		mg kg^−1^						
Manganese	0–10	124b^†^	120b	134ab	138ab	139ab	166a	16% ↓
	10–20	103ab	84b	88b	104ab	103ab	130a	21% ↓
	20–30	77a	74a	73a	67a	75a	94a	18% ↓
	30–60	85a	74a	76a	72a	71a	95a	11% ↓
Zinc	0–10	2.6b	1.7b	3.5b	1.8b	1.4b	6.0a	43% ↓
	10–20	2.0a	2.2a	2.3a	1.4a	3.1a	2.8a	11% ↑
	20–30	1.5a	0.5a	2.3a	0.2a	3.2a	1.1a	49%↓ 43%↑
	30–60	2.3a	1.2a	3.1a	0.4a	1.6a	1.0a	28%↓ 63%↑
Copper	0–10	1.1b	0.9b	0.7b	0.6b	0.6b	2.3a	53% ↓
	10–20	1.5b	1.9b	1.6b	1.5b	1.6b	2.6a	28% ↓
	20–30	2.4bc	2.2bc	2.1bc	1.7c	1.9bc	2.8a	15% ↓
	30–60	2.6ab	2.5ab	2.1b	2.2b	2.4b	3.1a	11% ↓
Boron	0–10	6.6a	6.2a	6.5a	6.7a	6.1a	0.0b	ND
	10–20	6.7a	6.2a	6.4a	6.5a	6.2a	0.0b	ND
	20–30	1.3a	0.7a	1.9a	0.2a	2.4a	1.1a	34%↓ 17%↑
	30–60	6.6a	6.3a	6.4a	7.0a	6.3a	0.0b	ND
pH	0–10	5.8b	5.4b	5.6b	5.1b	5.3b	6.8a	14% ↓
	10–20	6.1b	5.6b	5.9b	5.4b	5.6b	6.8a	11% ↓
	20–30	6.5ab	6.3b	6.5ab	6.4ab	6.5ab	7.0a	06% ↓
	30–60	6.6b	6.6b	6.8ab	6.6b	6.7ab	7.1a	05% ↓

^†^Means sharing the same letter within the rows are not significantly different at 5% level of significance.

^‡^Percentage calculated from the difference in the value of grass pasture (GP) and the highest value (if GP is greater) or the lowest value (if GP is lower) for the treatments within each soil depths, so that minimum deviation from the GP is calculated in either case. The downward and upward arrow indicates decline or incline from the soils of GP after cultivation, respectively. The column with both upward and downward in the same cell indicates that respective soil depth has some treatments that have greater value than GP and some treatments with lesser value than GP.

### Effect of tillage and N fertilization on micronutrients in wheat grain and straw

**Figure 3:**
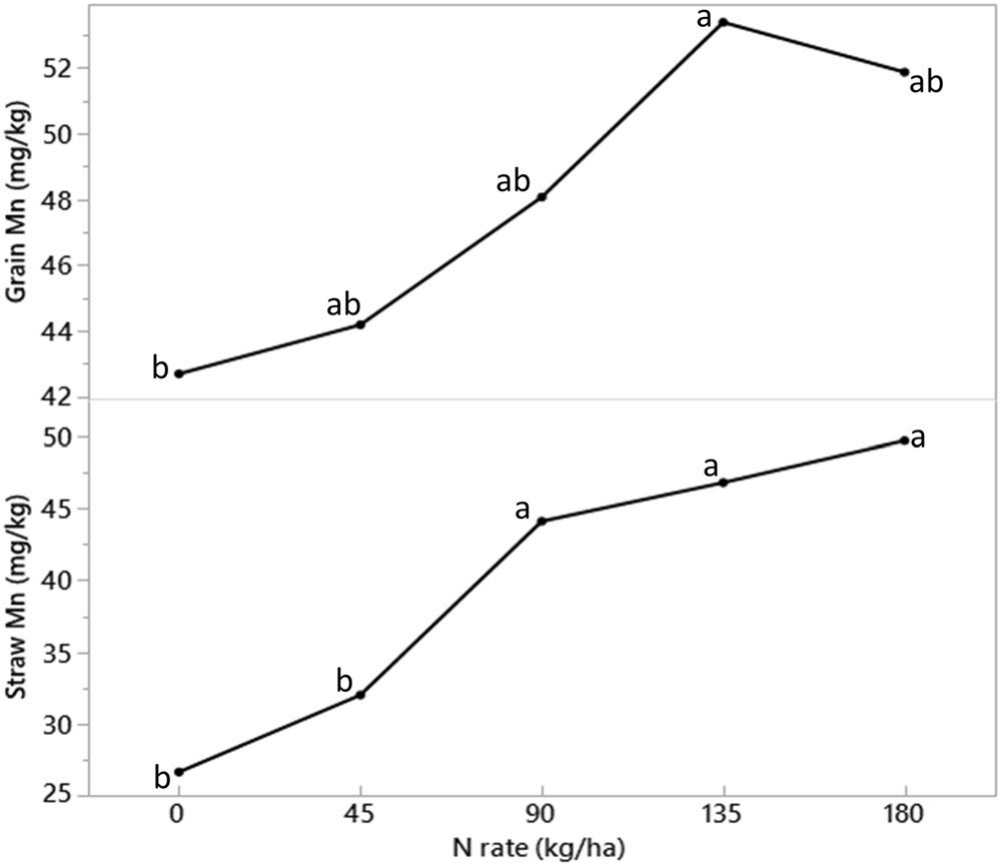
The long-term (75 years) effect of N application rates on wheat straw manganese (bottom) and grain manganese (top). Points sharing the same letters indicates no significant differences between treatments at 0.05 probability level.

The total Mn concentration in wheat straw was largely influenced by N application rate, whereas Cu, Fe, and B were affected by the interaction of tillage systems and year (Table 4). The Mn in straw increased linearly with increasing N application rates (Fig. 3). The concentrations of Mn in straw were 27, 32, 44, 47, and 50 mg kg^−1^ at the 0, 45, 90, 135, and 180 kg N ha^−1^, respectively. However, the concentrations of Cu, Fe, and B in the straw declined over the 20-year period (1995–2015) under all the tillage systems (Table 5). Except for the concentration of Mn, none of the micronutrients in wheat grain were affected by the treatments (Table 4). The Mn in grain increased with increasing N fertilizer rate up to 135 kg N ha^−1^ (Fig. 3). The concentrations of Mn in grain were 41, 44, 48, 54, and 52 mg kg^−1^ at the 0, 45, 90, 135, and 180 kg N ha^−1^ fertilization rates, respectively.

**Table 4:** ANOVA table for the main and interaction effects of tillage, year, and N rate on the concentration of total manganese (Mn), copper (Cu), zinc (Zn), iron (Fe), and boron (B) in wheat grain and straw.

Source of variation	Manganese	Copper	Zinc	Iron	Boron
**Wheat Straw**					
Tillage (T)	0.25	0.03	0.69	0.02	<0.01
Year (Y)	**<0.01**	<0.01	**<0.01**	<0.01	<0.01
N rate (N)	**<0.01**	0.94	0.61	0.15	0.12
T x Y	0.76	**<0.01**	0.94	**<0.01**	**<0.01**
N x Y	0.96	0.91	0.72	0.15	0.43
T x N	0.43	0.35	0.95	0.45	0.16
T x N x Y	0.55	0.09	0.98	0.97	0.26
**Wheat Grain**					
Tillage (T)	0.67	0.86	0.31	0.91	0.13
Year (Y)	**0.01**	**<0.01**	**<0.01**	**<0.01**	**<0.01**
N rate (N)	**0.03**	0.58	0.58	0.86	0.37
T x Y	0.71	0.41	0.69	0.55	0.11
N x Y	0.45	0.56	0.71	0.81	0.78
T x N	0.37	0.89	0.61	0.87	0.52
T x N x Y	0.27	0.88	0.56	0.83	0.76

Significant effects (p-value < 0.05) that require multiple means comparison are in bold.

**Table 5:** Interaction effect of tillage system and year on total concentrations of copper, iron, and boron accumulation in wheat straw.

Nutrients	Tillage	Year		
		1995	2005	2015
		mg kg^−1^		
Copper	Moldboard	2.0aB^†^	3.4aA	0.6aC
	Sweep	2.4aA	1.7bA	0.6aB
	Disk	1.7aA	2.3abA	0.5aB
Iron	Moldboard	48aA	52aA	32aB
	Sweep	50aA	30bB	29aB
	Disk	48aA	49aA	30aB
Boron	Moldboard	3.7aB	5.8aA	2.3aC
	Sweep	3.4aA	4.2abA	2.0bB
	Disk	3.4aAB	3.1abA	2.3aB

^†^Means followed by the same uppercase letter in a row indicate no significant differences between years for each tillage system and means followed by same lowercase letters in a column indicates no significant differences between tillage system in each year at 0.05 probability level.

## Discussion

### Tillage and N fertilization effect on soil micronutrients

Extractable Mn declined with depth. It is well documented that the availability of soil micronutrients is associated with SOM^1,6^. Our results agree with previous studies, which also reported increased concentrations of extractable Mn in the upper 10 cm soil depth under tillage that promoted accumulation of plant residues at the soil surface and resulted in poor soil mixing^1,20^. In this study, the DP and SW had lower volumes of soil mixing and left more residue on the soil surface compared to MP. Depth of soil disturbance was low under DP (10 cm), and SW (15 cm) compared to MP (23 cm) and consequently differed in the percentage of residue cover (OM) left on the top 10 cm soil.

The Mehlich III extractable Cu in the top 10 cm soil depth was higher under MP than under reduced tillage, which is in agreement with earlier studies^5,9^. Contrastingly, Mahler *et al*.^6^ found lower concentration of extractable Cu under MP than under reduced tillage, while Edwards *et al*.^8^ did not find a significant effect of tillage on soil Cu. Franzluebbers and Hons^9^ also reported increased Cu concentration until 30 cm depth whereas, in this study, extractable Cu increased beyond the 30 cm soil depth. Soil profile distribution of Cu can be explained by its interaction with SOM. Copper is bound to SOM and migrates into subsoil with SOM acting as a carrier, forming soluble metal-organic complexes^21^. The N fertilization reduced the Mehlich III extractable Cu in this study similar to Prasad and Power^22^. Inorganic N fertilization could reduce soil Cu by decreasing soil pH and increasing Al and Fe levels in soils^22^. The Fe and Al oxides and oxyhydroxides adsorb Cu tightly and consequently reduce the mobility of Cu in fertilized soils^22^.

### Soil micronutrients after 75 years of N fertilization and tillage versus grass pasture (GP)

Extractable Mn, Zn, and Cu and soil pH declined significantly after 75 years of N fertilization in the upper 10 cm soil depth at all tested N rates (Table 3). It is well-documented that N fertilization lowers soil pH, enhancing the availability of micronutrients^23,24^. It was evident in our study that the soil pH had decreased after 75 years of cultivation (Table 3) and significantly decreased in upper 20 cm soil surface (reported in another manuscript from the same experiment but with macronutrients^25^). However, this acidification did not increase micronutrient availability over the study period, suggesting that continuous removal through crop harvest and meager contributions from crop residue had depleted micronutrients in the soil. The other likely reason for the significant decline of extractable Mn, Cu, and Fe in the upper 10 cm soil would be due to the presence of a higher percentage of OM (crop residue) in the upper 10 cm soil than deeper in the soil profile. The availability of these nutrients in the soil solution decreases with higher OM, as these elements have a high affinity for OM resulting in stable bonding^24^.

### Effect of tillage and N fertilization on micronutrients in wheat grain and straw

Inorganic N fertilization increased the concentration of total Mn in wheat grain up to 135 kg N ha^−1^ application rate (Fig. 3). In contrast with these results, Hamnér *et al*.^26^ reported that N fertilization did not influence grain Mn in their study; however, they found increased concentrations of Fe, Zn, and Cu in the wheat grain as a function N fertilization. The relationship between N fertilization and micronutrients is unclear, but previous studies have indicated a correlation of N to the movement of micronutrients within plants^27,28^.

## Conclusion

The findings of this study are significant for a sustainable dryland winter wheat-fallow cropping system. The results provide important insight into the impact of long-term tillage and inorganic N fertilization (75-years) on the distribution of micronutrients (Mn, Cu, Fe, Zn, and B) in soil and wheat. The study demonstrated the declining trend in the concentrations of extractable Mn, Cu, and Zn in cultivated soil (cultivation effect) when compared to the undisturbed grass pasture plot. It is evident that continuous cultivation with N fertilization and tillage may significantly reduce concentrations of plant essential nutrients over time. We found that disk plow tillage and high N application rates were better than other treatments studied. However, nitrification derived acidity must be considered and should be regularly monitored. Integration of organic amendments and inorganic nitrogen fertilizer application in nutrient management strategy may help to increase micronutrients in soil and wheat in a long-term as organic amendments are known to enhance nitrogen and micronutrients availability without acidifying the soil. A long-term study is needed to warrant the benefits of integrating organic amendments and inorganic N on micronutrients availability over time in the drylands of the PNW.

## Materials and Methods

### Study sites and experimental design

The study was conducted at one of the ongoing long-term experiments (LTE) of the Columbia Basin Agriculture Research Center (CBARC), near Pendleton, OR (45°42′N, 118°36′W, elev. 438 m.a.s.l.). This LTE was established in 1940 on a a well-drained Walla Walla silt loam soil (coarse-silty, mixed, superactive, mesic Typic Haploxeroll) with a 2–4% slope. The mean annual temperature is 10 °C, and ranges from −1 °C in January to 21 °C in July. Mean annual precipitation is 437 mm. The top 30 cm soil depth contains 20% clay, 68% silt, and 1.1% organic C, and has 16 cmolc kg^−1^ cation exchange capacity (CEC).

The experimental plot is a randomized block, split-plot tillage and fertility experiment with three replications under dryland winter wheat-14 months fallow (WW-F) cropping system. Each block was divided into three main plots as tillage treatments and each main plot was divided into five subplots as N fertilization treatments. The three tillage treatments were moldboard plow (MP), disk plow (DP) and sweep (SW) with the size of 35 by 40 m each. Subplots comprised of five N fertilization rates (0, 45, 90, 135, and 180 kg N ha^−1^) and were 5.8 by 40 m in size. During late March to early April, primary tillage was performed in the fallow plots on the stubble left undisturbed since wheat harvest. The three tillage treatments differed in tillage equipment, surface residue cover at the time of seeding, and tillage depth. The percentage of residue cover left by MP, SW, and DP were 7%, 43%, and 34% respectively, and the tillage depths were 23 cm, 15 cm, and 10 cm, respectively. The MP is a soil inversive tillage whereas DP and SW are non-soil inversive tillage. Therefore, the MP is considered conventional tillage, and the SW and DP are considered reduced/conservation tillage in this study.

A nearby grass pasture (GP) plot, undisturbed since 1931, was used as reference/baseline for this study to compare changes in treatments over time. The dominant grasses in this pasture are blue-bunch wheatgrass (*Agropyron spicatum* L. Pursh) and Idaho fescue (*Festuca idahoensis* L. Elmer).

### Field operations and soil sampling

After wheat harvesting in late July, the stubble was left undisturbed until primary tillage operations in late March. Plots were rod weeded two to four times between April and October to control weeds. During the first week of October, urea ammonium nitrate fertilizer was added to the top 10 cm soil using Viper Coulter (Yetter Manufacturing Inc. Colchester, IL). A week after N fertilization, wheat was seeded at the rate of 72 ± 5 kg seed ha^−1^ in 25 cm rows spacing. A JD8300 drill (Deere and Company, Moline, IL) was used for wheat seeding before 2002, and thereafter a Case IH 5300 disk drill (Klamath Basin Eq. Inc. Klamath Falls, OR) was used. The seed variety was Malcolm during the 1995–2005 period, and Stephens after that. Both were semi-dwarf varieties of winter wheat. Weeds were controlled using herbicides during the growing season.

The soils were sampled by compositing the cores of north-central and south-central of each plot. Wheat grain and straw samples were collected from the center of the plot after the wheat harvest. The soils were sampled from four depths (0–10, 10–20, 20–30, and 30–60 cm) using a truck-mounted Giddings Hydraulic Probe (Giddings Machine Company, Inc., Windsor, CO) and a steel sampling tube (internal diameter 3.6 cm). In this study, the soil and plant samples from 1995 (archived samples), 2005 (archived samples) and 2015 cropping season were used. The ground soil samples were processed and analyzed at the Central Analytical Laboratory (CAL, Oregon State University). The Mehlich III method^29^ was used to extract available Mn, Cu, Fe, B, and Zn from the soil samples, and a dry ash method^30^ was used to extract the total concentration of these nutrients from the grain and straw samples. An inductively coupled plasma-optical emissions spectroscopy (ICP-OES, Model #2100 DV, Waltham, Massachusetts, USA) was used to determine the nutrients in soil and plant tissue extracts. Soil pH data were provided by the CBARC, and were determined with a pH electrode using 10 g samples in a 1:2 soil to 0.01 M CaCl_2_ solution.

### Statistical analysis

A split-plot design analysis was used to test the effect of the treatments on the concentration of Mn, Cu, Fe, B, and Zn using the mixed model procedure in JMP^©^ version 13^31^. Tillage system, N rates, and soil depths were considered the fixed effects while analyzing soil micronutrients. We didn’t observe significant differences in soil micronutrients as a function of year and its interaction, therefore the analysis was done using the 2015 data only. Tillage system, N rates, and year were considered the fixed effects for tissue analysis. Replications and their interactions were considered the random effects in both the soil and tissue analysis. Multiple comparisons with Tukey methods were performed to determine differences in nutrients and letter groupings were generated using a 5% level of significance.

Soil pH data were converted to H^+^ concentration (µmol L^–1^) before ANOVA was performed. The pH scale is a logarithmic and small differences in pH represent large differences. However, the mean comparisons of soil pH represent the original pH data.
